# Integrated analysis of the heterogeneous microarray data

**DOI:** 10.1186/1471-2105-12-S5-S3

**Published:** 2011-07-27

**Authors:** Sung Gon Yi, Taesung Park

**Affiliations:** 1 Department of Epidemiology and Biostatistics, Case Western Reserve University, Cleveland, OH, USA; 2 Department of Statistics, Seoul National University, Seoul, South Korea

## Abstract

**Background:**

As the magnitude of the experiment increases, it is common to combine various types of microarrays such as paired and non-paired microarrays from different laboratories or hospitals. Thus, it is important to analyze microarray data together to derive a combined conclusion after accounting for heterogeneity among data sets. One of the main objectives of the microarray experiment is to identify differentially expressed genes among the different experimental groups. We propose the linear mixed effect model for the integrated analysis of the heterogeneous microarray data sets.

**Results:**

The proposed linear mixed effect model was illustrated using the data from 133 microarrays collected at three different hospitals. Though simulation studies, we compared the proposed linear mixed effect model approach with the meta-analysis and the ANOVA model approaches. The linear mixed effect model approach was shown to provide higher powers than the other approaches.

**Conclusions:**

The linear mixed effect model has advantages of allowing for various types of covariance structures over ANOVA model. Further, it can handle easily the correlated microarray data such as paired microarray data and repeated microarray data from the same subject.

## Background

Microarray technology has important applications in pharmaceutical and clinical research. For example, microarrays can be used to identify tumor-related genes and targets for therapeutic drugs. In microarray experiments, the identification of differentially expressed genes (DEG) is an important issue. Statistical test procedures have served as useful tools for identifying the DEGs which can be candidate genes for a specific disease or can be used for the further analysis such as clustering analysis and gene regulatory network construction.

As the cost of producing microarrays has become lower costs and the importance of replication in microarray experiments has been demonstrated by many researchers [1], replicated microarrays are commonly used in microarray experiments. In order to handle replicated microarrays, many statistical test procedures have been developed, such as *t*-statistics, to identify DEGs between two groups [2]. The analysis of variance (ANOVA) model approach was proposed to identify DEGs among multiple groups [3]. In addition, many statistical models have been proposed to identify the DEGs on replicated microarrays [4-11].

When the magnitude of a microarray experiment increases, it is common to use the same type of microarrays from different laboratories or hospitals. Thus, it is important to analyze microarray data together to derive a combined conclusion after accounting for the differences. Recently, statistical approaches based on meta-analysis have been proposed in order to combine independent and heterogeneous microarray studies [12-15]. In these approaches, microarrays were classified into several independent groups and integration methods to analyze microarray data sets from different laboratories were proposed. The key idea of meta-analysis is to combine summary statistics from each study in which significant levels (p-values) and effect sizes are commonly used as summary statistics. Meta-analysis requires data be homogeneous within the data set. When there are microarray-specific covariates such as gender and smoking status, meta-analysis can be less effective.

Shen *et al*. (2004) introduced the probability of expression (POE) and proposed a method to estimate the POE using MCMC [16]. The POE is the scale-free measure transformed from raw gene expression defined by the difference between probabilities of over- and under-expressed gene expression. Using the POE, the gene expressions of heterogenous microarray experiments can be uniquely scaled from -1 and 1 and combined easily. Choi *et al*. (2007) proposed EM algorithm to estimate the POE instead of MCMC, which can reduce the estimation time of the POE [17]. Standardized POE can combine multiple microarray data sets, however, the POE method can be more efficient when the microarray-specific covariates are applied.

Park *et al*. [18] proposed a two-stage ANOVA model approach for the integrated analysis, which uses the ANOVA model with controlling variables for additional variability of heterogeneous microarray studies. The usual ANOVA model was extended to account for an additional variability resulting from many confounding variables. When variability among data sets is relatively small, the ANOVA model is effective. Otherwise, the ANOVA model is not recommended. Further, when the microarrays are correlated, the ANOVA model cannot handle such correlation appropriately, because it requires the independence of samples. Therefore, correlated microarray data can violate the assumption of the ANOVA model and thus the extended model to allow for various types of covariance structure of errors is needed.

In this paper, we propose the linear mixed effect (LMe) model for the integrated analysis of the heterogeneous microarray data sets. The LMe model contains various random effects which effectively account for the heterogeneous variability in the data from many different sources. Further, the LMe model has advantages of allowing for various types of covariance structures over meta-analysis and ANOVA model approaches. Thus, it can handle easily the correlated microarray data such as paired and non-paired microarray data. The proposed method is illustrated using the liver cancer microarray data sets obtained from three different hospitals [14].

## Materials and methods

Four independent microarray data sets were generated from three hospitals using two different chips [15]. The first chip, *C*_1_, contains 10,336 human cDNA probes that were verified by single pass sequencing. The second chip, *C*_2_, contains 10,368 human cDNA probes. Two chips shared the common 9,984 cDNA probes. The chips were cDNA chips with two-colors, where the way of labeling samples and controls is described in Choi *et al*. (2004). A further detailed description of the chips has been uploaded to the Gene Expression Omnibus (GEO) site (http://www.ncbi.nlm.nih.gov/geo/) with GEO accession number GPL2911.

The chip type (1 and 2), labeling scheme, hospital and number of samples are shown in this table. Here, the data were normalized by locally weighted scatterplot smoothing (LOWESS; Cleveland, 1979). For LOWESS normalization, the value of the span parameter was 0.75 and the tricubic function was used as a weight function. For robustness analysis, Tukey’s biweight function was used [18]. Hepatocellular carcinoma (HCC) and adjacent control (normal) samples were obtained with informed consent from patients at three hospitals. All the HCC samples were hepatitis B virus (HBV) positive. Sample preparation, microarray hybridizations, and fluorescence signal acquisitions were carried out independently at each institution according to similar but not identical experimental protocols and laboratory conditions.

Table 1 summarizes the descriptive information for the microarray experiment. Thirty two microarrays were produced from 17 patients in Hospital A yielding data set D1. A pair of microarrays for each patient were produced from two cDNA samples: one from the HCC sample and the other from the control(normal) sample. In Hospital A, fifteen pairs of microarrays and two individual microarrays were produced. Forty six microarrays were produced from 23 patients in Hospital B yielding data set D2. Fifty five microarrays were produced from 43 patients in Hospital C. Only twelve pairs of microarrays and 37 individual microarrays were produced. Chip *C*_2_ was used only on 21 microarrays from 13 patients in Hospital C. Other microarrays were produced by Chip *C*_1_. Microarrays from Hospital C were divided into two data sets (D3 and D4) according to the chip type. All microarray data were obtained using the reference design with the placenta as the reference.

**Table 1:** Descriptive information for the liver cancer microarray data

Data set ID	Hospital	Chip type	Number of paired samples		Number of non-paired samples		Total number of samples
				
			tumor	control	tumor	control	
D1	A	*C*_1_	15	15	1	1	32
D2	B	*C*_1_	23	23	0	0	46
D3	C	*C*_1_	4	4	25	1	34
D4	C	*C*_2_	8	8	4	1	21

### The LMe models

Suppose there are *H* multiple data sets denoted by *h* = 1, …, *H.* There are *n_h_* patients for the *h*th data set. In our study, *H* = 4 and treatment groups consist of two levels denoted by *k* = *T*, *C*, where one (*k* = *T*) is the tumor tissue group and the other (*k* = *C*) is the control tissue group. For the paired observations, *k* has two values *T* and *C.* For the non-paired observation, *k* has only one value of *T* or *C.* Assume there are *N* common probes on each chip for all data sets. We denote genes by *l* (= 1,…, *N*)*.*The linear mixed effects (LMe) model consists of both fixed effects and random effects. The LMe model for the *l*th gene is given by

**Y**_*hil*_ = **X**_*hil*_***β***_*l*_ + **Z**_*hil*_**b**_*hil*_ + ***ε***_*hil*_,

*h* = 1, …, *H*, *i* = 1, …, *n_h_*, *l* = 1, …, *N*, (1)

where **Y***_hil_* is a response vector for the *i*th subject (patient) of the *h*th data set, ***β****_l_* is the fixed effect parameter vector, **b***_hil_* is the random effect parameter vector, and **ε***_hil_* is the error vector. Random effects and errors are assumed to be independent and normally distributed:

**b***_hil_* ~ *N*(**0**, **Φ***_l_*),  **ε***_hil_* ~ *N*(**0**, **I***σ*^2^). (2)

The variance of random effects **Φ***_h_* can have several forms. When the off-diagonal terms are zero, then the random effects are uncorrelated. Otherwise, they are correlated. By allowing different forms of **Φ***_h_*, we can model variability among samples efficiently. When there are no random effects, say **Z***_hil_* = 0, the LMe models become equivalent to the ANOVA models.

For the liver cancer data, there are three fixed effects: treatment, hospital, and chip type. The LMe model is given by following equation:(3)

where *l* = 1,…, 9984, *h* = 1,…, 4, *β_Tl_* represents the treatment effect of differences between tumor tissue and control tissue, *β_Cl_* represents the effect of differences between two chips, and two parameters, *β*_*H*_1_*l*_ and *β*_*H*_2_*l*_, represent the effect of differences among hospitals.

### Types of covariance structure

The most general form of covariance matrix in the LMe models assumes the covariance matrix of gene expressions within each data set is unstructured and differs among data sets. However, this covariance matrix requires many parameters to be estimated, which could result in a possible loss of power. Therefore, we need to consider simplified forms of the covariance matrices of **b***_hil_*. We consider four types of covariance forms for the integrated analysis of microarray data. For simplicity, we start with the case when the data consist of all paired observations.

#### Paired microarrays

1. Type 1: General form Covariance matrix of **b***_hil_*:

2. Type 2: One common unstructured covariance matrix for all data sets Covariance matrix of **b***_hil_*:

3. Type 3: Compound symmetry covariance matrix with different variance parameter for each data set **b***_hil_* has only one component *b_hil_* and its variance is given by

4. Type 4: One common compound symmetry covariance matrix with the same variance parameter for all data sets **b***_hil_* has only one component *b_hil_* and its variance is given by

Type 2 assumes the covariance matrix of gene expressions within each data set is unstructured like Type 1 but it is the same over the data sets, which is a simplified form of Type 1. Type 3 assumes each covariance matrix within the data set is compound symmetric and differs over the data sets. Type 4 is simplified version of Type 3 assuming the same covariance matrix over the data sets.

For all types of covariance structure, the variance of **Y***_hil_* is given by

**Y***_hil_* = *Var*(**b**_hil_) + **I***σ*^2^.

#### Non-paired microarrays

For the non-paired observation, the dimension of **Y***_hil_* becomes one. The LMe allows only a scalar random effect. That is, **b***_hil_* has only one component *b_hikl_*. For example, Type 1 assumes(4)

### Tests

LMe model parameters can be estimated via maximum likelihood estimation. The DEGs can be identified by testing whether *β_Tl_* = 0 or not. LMe models also suffer from the multiple testing problem. We apply the FDR adjustment method proposed by Benjamini *et al.*[*19*].

## Results

### Analysis of the liver cancer microarray data

We applied the integrated analysis using LMe models, two-stage ANOVA model, and meta-analysis to liver cancer data. The LMe model is given in Equation 3. We fit this LMe model by assuming that **b***_hil_* has the covariance structure of Types 1 to 4. These four models are denoted by M1, M2, M3, and M4, respectively. The last LMe model M5 assumes no random effects and is expected to provide similar results to the two-stage ANOVA model.

Table 2 shows the number of DEGs for the given FDRs. A much larger number of genes were identified by integrated analyses. When FDR is controlled by 5%, the number of DEGs on each individual microarray data set D1 to D4 are only 0, 38, 0, and 0, respectively [18]. However, the number of identified genes by meta-analysis, ANOVA model, five LMe models are 197, 145, 214, 543, 589, 375, and 114, respectively. The number of significant genes varied across methods. The smallest number of genes was selected by the LMe model M5 which requires the independent assumption between the paired microarrays. A similar number of genes was selected in meta-analysis and ANOVA model using the permutation tests. Much larger number of genes were selected by LMe models M1, M2, M3, and M4. The largest number of genes was identified by M3 implying that M3 is more powerful method than other LMe models. However, the results may contain false positive errors. In Subsection Simulation Study, we investigate this issue through a simulation study and show that M3 controls Type I error rate well.

**Table 2:** Genes that are identified as differentially expressed when FDR is controlled 1%, 5%, 10%, and 20%, respectively

FDR	Meta analysis	Two-stage ANOVA	LMe				
			
			M1	M2	M3	M4	M5
1%	57	46	119	184	205	124	37
5%	197	145	214	543	589	375	114
10 %	303	203	339	879	978	740	181
20 %	478	336	585	1500	1761	1323	342

**Figure 1:**
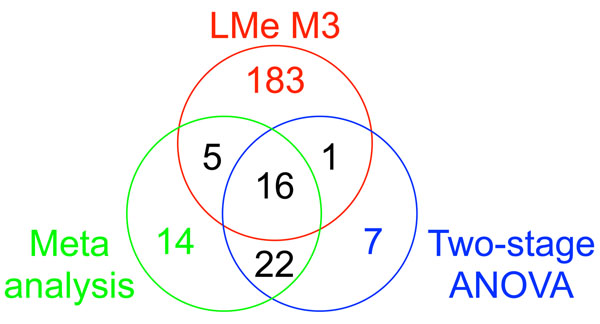
Number of genes that are identified by meta-analysis, two-stage ANOVA, and LMe model M3 when FDR is controlled by 1%

Now, we focus on comparing the results. For simplicity, we consider LMe model M3 only for summarizing the LMe models. Figure 1 shows the number of the significant genes identified by meta-analysis, two-stage ANOVA model, and LMe model M3 when FDR is 1%.

The number of common genes selected by all three methods was only 16. Among them 9 genes are known and Table 3 summarizes their characteristics. The number of common genes selected by two-stage ANOVA model and meta-analysis was 22. On the other hand, the numbers of the common genes selected by LMe with others are small.

**Table 3:** Common genes detected by meta-analysis, two-stage ANOVA model, and LMe M3 model when FDR is controlled by 1% (9 known genes)

Unigene ID	Description
Hs.82084	Integrin beta 3 binding protein (beta3-endonexin) (ITGB3BP), mRNA
Hs.514	Cyclin H (CCNH), mRNA
Hs.167529	Cytochrome P450, subfamily IIC (mephenytoin 4-hydroxylase), polypeptide 9 (CYP2C9), mRNA
Hs.117367	Solute carrier family 22 (organic cation transporter), member 1 (SLC22A1), mRNA
Hs.54900	Serologically defined colon cancer antigen 1 (SDCCAG1), mRNA
Hs.80756	Betaine-homocysteine methyltransferase (BHMT), mRNA
Hs.8765	RNA helicase-related protein (RNAHP), mRNA
Hs.755990	Haptoglobin (HP), mRNA
Hs.35101	Proline-rich Gla (G-carboxyglutamic acid) polypeptide 2 (PRRG2), mRNA

The number of genes identified only by M3 was 183 in the Figure 1 Some genes have been found to be related with liver disorders (BChE, C6, C9, CAP2, CDKN2A, CtBP, Cul4A, Gab1, Id1, NTRK1, PSG1, and PSMG). HChE was shown to exhibit highly elevated aryl acylamidase activity (AAA). The absolute levels of AAA were increased as BChE activity decreased while deviating from normal samples and such deviation was directly proportional to the severity of the liver disorder [20].

C6 is a component of the complement system, which plays an important role as a humoral effector system during inflammation and infection, and consists of more than 25 components, including regulatory proteins. C6 was shown to late-acting complement proteins that participate in the assembly of the membrane attack complex, which causes cell lysis by the formation of pores in the cell membrane of certain microorganisms. [21]. C9 was related to the medication of tumor PDT by photosensitizer Photofrin using mouse Lewis lung carcinoma (LLC) model [22]. Cyclase-associated protein 2 (CAP2) was listed as an up-regulated gene in early hepatocellular carcinoma (HCC) [23]. CDKN2A was reported to be differentially regulated by methylation between normal tissue and HCC. Low levels of methylation in normal tissue and adjacent tissue but high levels in HCC [24]. C-terminal binding protein (CtBP) was reported to relate with INK4A/ARF tumor suppressor gene. The INK4A/ARF tumor suppressor locus is frequently inactivated in HCC. Inhibition of cell invasion by p19Arf was dependent on its C-terminal binding protein (CtBP) [25]. The Cul4A gene is amplified in human breast and liver cancers, and loss-of-function of Cul4 results in the accumulation of the replication licensing factor CDT1 in Caenorhabditis elegans embryos and ultraviolet (UV)-irradiated human cells [26].

Gab1 was reported to be related with hepatic insulin action. Deletion of Gab1 in the liver leads to enhanced glucose tolerance and improved hepatic insulin action. It was also shown that association of Gab1 adaptor protein and Shp2 tyrosine phosphatase is a critical event at the early phase of liver regeneration [27,28]. Id1 was identified as TGF-*β*/ALK1/Smad1 target gene in HSCs and represents a critical mediator of transdifferentiation that might be involved in hepatic fibrogenesis. Transforming growth factor (TGF)-*β* is critically involved in the activation of hepatic stellate cells (HSCs) that occurs during the process of liver damage, for example, by alcohol, hepatotoxic viruses, or aflatoxins [29,30]. NTRK1 was reported to be a favorable neuroblastoma (NB) genes. NB is a common pediatric solid tumor that exhibits a striking clinical bipolarity: favorable and unfavorable. High-level expression of NTRK1 predicts favorable NB outcome and inhibits growth of unfavorable NB cells [31]. PSG1 was reported to an up-regulated gene in a fetal liver [32]. PSMG was reported to significantly elevated expression in HCC [33].

### Simulation study

In order to evaluate the proposed methods, we simulated the two sets of microarray data and then performed the integrated analysis by using the proposed LMe method as well as other methods. For simplicity, we assume the log-transformed ratio of two intensities are normally distributed. To mimic the liver cancer microarray data, we assume that a pair of microarrays are obtained from the same patient. The first microarray data set consists of 60 microarrays from 30 patients and the second data set consists of another 60 microarrays from 30 patients. Suppose that two microarrays from the same patient are from different groups, say from tumor and control tissues. The main objective of the analysis is to identify the DEGs between two groups.

We assume the number of genes in each microarray is 30 among which the six genes (20%) are truly differentially expressed: three genes are over-expressed and the other three genes are under-expressed in the tumor tissue. The simulation model is given by

where *β_Dl_* represents a fixed effect of the difference between two data sets and *β_Tl_* represents a fixed effect for difference of expression levels between tumor and control tissues. The values of *β_Tl_*s are 1.5 for *l* = 1, ⋯, 3, and -1.5 for *l* = 4, ⋯, 6, respectively, and zero for *l* = 7, …, 30. The values of *β_Dl_* are randomly determined by generating random variables from the standard normal distribution. Errors are also generated from the normal distribution with mean 0 and variance *σ*^2^ = 0.5^2^.

For the random effect *b_hikl_* we assume three types of covariance matrix corresponding to Types 1, 2, and 3 defined in Section Types of Covariance Structure. For Type 1, the covariance matrix **b***_il_*=(*b*_1_*_iTl_*, *b*_1_*_iCl_*, *b*_2_*_iTl_*, *b*_2_*_iCl_*)*^T^* is given by(5)

We set values of parameters as *σ*_11_ = 1, *σ*_12_ = 2, *σ*_21_ = 1.5, and *σ*_22_ = 2.5. In addition, the correlation parameter between tumor and control tissues are set as 0, 0.2, and 0.4. For Type 2, two variance parameters are set as *σ*_1_ = 2.5 and *σ*_2_ = 1, and the correlation parameters are set as 0, 0.2, and 0.4 as Type 1. Finally, for Type 3, two variance parameters are set as *σ*_1_ = 2.5 and *σ*_2_ = 1. For the detailed information of the covariance structure, see Table 4.

**Table 4:** Setting for random effects *b_hik__l_*

Type	random effects of subject	covariance of random effects
1		

2		

3		

For the simulated data sets, we perform the analyses using the meta-analysis, the two-stage ANOVA model and five LMe models. We fit this LMe model by assuming that **b***_il_* has the covariance structure of Types 1 to 4. These four models are denoted by M1, M2, M3, and M4, respectively. The last LMe model M5 is the one assuming no random effects, which is expected to provide similar results to the two-stage ANOVA model.

Table 5 shows powers and FDRs from 1,000 simulated data sets. The threshold value *q* was 0.05. Genes having ordered *q* values smaller than 0.05 were identified as DEGs. Note that there are 6 true significant genes and 24 null genes. The empirical FDR values were computed as the number of false significant genes from 24 null genes divided by the total number of significant genes. The empirical power was computed as the number of significant genes among the 6 true genes divided by 6.

**Table 5:** Power and FDR of methods under simulated data of Types 1, 2, and 3 covariance structures when FDR was controlled by 0.05

Type	*ρ*		Meta analysis	Two-stage ANOVA	LMe				
					
					M1	M2	M3	M4	M5
1	0	Power	0.2087	0.1983	0.2770	0.2073	0.2610	0.2273	0.2240
		FDR	0.0740	0.0600	0.1150	0.0730	0.0863	0.0746	0.0807
	
	0.2	Power	0.1863	0.1683	0.3493	0.2570	0.2847	0.2607	0.1903
		FDR	0.0345	0.0307	0.0958	0.0666	0.0707	0.0668	0.0371
	
	0.4	Power	0.1543	0.1403	0.4783	0.3950	0.4007	0.3953	0.1580
		FDR	0.0170	0.0186	0.0718	0.0558	0.0573	0.0557	0.0104

2	0	Power	0.1453	0.1423	0.3650	0.3067	0.3093	0.3073	0.1570
		FDR	0.0224	0.0251	0.0920	0.0564	0.0569	0.0563	0.0248
	
	0.2	Power	0.1290	0.1347	0.3373	0.2867	0.2867	0.2867	0.1450
		FDR	0.0203	0.0098	0.0858	0.0591	0.591	0.0591	0.0247
	
	0.4	Power	0.1490	0.1497	0.3700	0.3150	0.3170	0.3150	0.1503
		FDR	0.0283	0.0323	0.1091	0.0680	0.0676	0.0680	0.0363

3		Power	0.1517	0.0010	1.000	1.0000	1.0000	1.0000	0.0043
		FDR	0.0000	0.0000	0.0712	0.0455	0.0455	0.0455	0.0000

When *ρ* was zero, powers and FDRs showed very consistent results for all methods, although the variances of tumor tissue and control tissue are assumed to be different. This means all methods perform similarly when the correlation between tumor and control tissues does not exist.

Table 5 summarizes the simulation results for Type 1 covariance matrix. In general, meta-analysis, two-stage ANOVA model analysis, and M5 provided similar results in powers and FDRs. On the other hand, other LMe models provided quite different results. For example, the FDRs tend to be larger but maintain 5% level approximately except for M1. Powers of LMe models tend to be much larger than meta-analysis and two-stage ANOVA model analysis. Among the five LMe models, M1 and M5 provide distinct results from the other three models M2, M3, and M4.

It is interesting to note that the performance of each method depends on the value of *ρ*. For meta-analysis, two-stage ANOVA, and M5, the powers decrease as *ρ* increases. On the other hand, the powers of LMe models M1 to M4 increase. These tendencies illustrate that meta-analysis and two-stage ANOVA do not handle correlations efficiently as LMe models do.

FDRs of LMe models, M2, M3, and M4 are slightly larger than 0.05. However, the FDR of M1 is much larger than 0.05, especially when *ρ* is close to zero. Thus, M1 is not appropriate to use when there is no correlation between tumor and control tissues.

Table 5 also summarizes the simulation result for the Type 2 covariance matrix showing similar patterns with those of Type 1 except that the results are less sensitive to *ρ*. In summary, meta-analysis, ANOVA model analysis, and M5 provided similar results in powers and FDRs. On the other hand, other LMe models provided quite different results. Among the five LMe models, M1 and M5 provided distinct results from the other three LMe models. The powers of LMe models M1 to M4 are larger than meta-analysis, ANOVA, and M5. Although M1 has the largest power, it also shows the largest FDR.

Finally, Table 5 also summarizes the simulation result for the Type 3 covariance matrix. Though correlation parameter *ρ* was not considered in this case, the correlation between tumor tissue and control tissue of same patient was assumed by the shared random parameter *b_hil_*. The results of simulated data under Type 3 are quite different from those obtained from Types 1 and 2. That is, all LMe models, M1, M2, M3, and M4 show extremely good performance. The powers are all 1 and FDRs are well-controlled around 0.05. LMe models work very well for this high correlation case. On the other hand, meta-analysis, ANOVA, and M5 performed worse. Among these, meta-analysis showed a slightly better performance. It is probably due to the fact that the meta-analysis allows different variances between two data sets, while others do not.

## Discussion

The LMe model is much more flexible than meta-analysis. One of the main limitations of meta-analysis is that it cannot handle the sample-specific covariates appropriately. Effect-size is simply the standardized mean difference between tumor tissue and control tissue [14]. Meta-analysis requires data are homogeneous within the data set, although data may be heterogeneous across data sets. For example, when there is sex information in data, the effect-size statistic cannot account for the sex effect directly. On the other hand, LMe models can handle individual specific covariates easily. In microarray studies, many researchers want to account for the individual characteristics in the analysis by including them as controlling variables. For example, the covariates such as age, sex, tumor stage, and weight might be important controlling variables. These covariates are usually sample-specific and differ across samples.

When there are no random effects, the LMe models become equivalent to the ANOVA models. The heterogeneity among data sets is only represented by the fixed effects. When heterogeneity among data sets is small, the ANOVA model can easily handle the variability among the data sets. However, when data sets have high variability and contain the correlated data, the addition of only fixed effects may not be satisfactory. In this case, the LMe model is more appropriate to analyze data sets, because it can model the heterogeneous variance and correlation structure more appropriately. The proposed LMe model is capable of handling heterogeneous covariance structures by allowing for various random effects.

When the data set contains paired and non-paired microarrays simultaneously, both meta-analysis and ANOVA model approaches cannot handle them appropriately. For example, the meta-analysis and the ANOVA analysis treated paired microarrays as independent microarrays. On the other hand, the proposed LMes can handle appropriately the correlation between the paired microarrays.

Finally, note that the proposed LMe model is valid when the normality assumption holds. We do not expect this assumption to hold for real microarray data. However, we expect the assumption is decreased when sufficiently large number of microarrays were combined. In future studies, we will develop permutation tests for the LMe models which do not require any distributional assumption.

## Conclusion

We proposed the LMe model for the integrated analysis of microarray data to identify DEGs in the presence of many controlling variables. We analyzed the liver cancer microarray data set and simulated microarray data to evaluate the performance of the integration methods. LMe models except M1 maintained FDRs approximately. Powers of LMe models except M5 tended to be much larger than meta-analysis and two-stage ANOVA model analysis. These tendencies illustrated that meta-analysis and two-stage ANOVA do not handle correlations efficiently as LMe models do.

## Competing interests

The authors declare that they have no competing interests.

## Authors' contributions

Sung Gon Yi performed the statistical analysis and drafted the manuscript. Taesung Park conceived of the statistical method for analysis and helped to draft the manuscript. All authors read and approved the final manuscript.
